# Occurrence of tuberculosis among people exposed to cattle in Bangladesh

**DOI:** 10.1002/vms3.1178

**Published:** 2023-06-16

**Authors:** Shamim Sarkar, Najmul Haider, Ariful Islam, Muhammad Belal Hossain, Kamal Hossain, Mohammad Khaja Mafij Uddin, Arfatur Rahman, Syed Sayeem Uddin Ahmed, Sayera Banu, Zeaur Rahim, James D. Heffelfinger, Nord Zeidner

**Affiliations:** ^1^ Programme on Emerging Infections Infectious Diseases Division, International Centre for Diarrhoeal Disease Research, Bangladesh Dhaka Bangladesh; ^2^ School of Life Sciences Keele University Keele Staffordshire UK; ^3^ Centers for Disease Control and Prevention (CDC) Atlanta Georgia

**Keywords:** Bangladesh, cattle market, cattle, chest disease hospital, *Mycobacterium bovis*, *Mycobacterium tuberculosis*, slaughterhouse, tuberculosis

## Abstract

**Background:**

Tuberculosis (TB) has been an important public health concern in Bangladesh. The most common cause of human TB is *Mycobacterium tuberculosis*, while bovine TB is caused by *Mycobacterium bovis*.

**Objective:**

The objective of this study was to determine the frequency of TB in individuals with occupational exposure to cattle and to detect *Mycobacterium bovis* among cattle in slaughterhouses in Bangladesh.

**Methods:**

Between August 2014 and September 2015, an observational study was conducted in two government chest disease hospitals, one cattle market, and two slaughterhouses. [Correction added on 27 June 2023, after first online publication: In the preceding sentence, the year “2014” has been added after the word “August”.] Sputum samples were collected from individuals who met the criteria for suspected TB and had been exposed to cattle. Tissue samples were collected from cattle that had low body condition score(s). Both humans and cattle samples were screened for acid‐fast bacilli (AFB) by Ziehl–Neelsen (Z‐N) staining and cultured for Mycobacterium tuberculosis complex (MTC). Region of difference (RD) 9‐based polymerase chain reaction (PCR) was also performed to identify *Mycobacterium spp*. We also conducted Spoligotyping to identify the specific strain of *Mycobacterium spp*.

**Results:**

Sputum was collected from a total of 412 humans. The median age of human participants was 35 (IQR: 25–50) years. Twenty‐five (6%) human sputum specimens were positive for AFB, and 44 (11%) were positive for MTC by subsequent culture. All (*N* = 44) culture‐positive isolates were confirmed as *Mycobacterium tuberculosis* by RD9 PCR. Besides, 10% of cattle workers were infected with *Mycobacterium tuberculosis* in the cattle market. Of all TB (caused by Mycobacterium tuberculosis) infected individuals, 6.8% of individuals were resistant to one or two anti‐TB drugs. The majority of the sampled cattle (67%) were indigenous breeds. No *Mycobacterium bovis* was detected in cattle.

**Conclusions:**

We did not detect any TB cases caused by *Mycobacterium bovis* in humans during the study. However, we detected TB cases caused by *Mycobacterium tuberculosis* in all humans, including cattle market workers.

## Graphical abstract

Overall, 11% people (involved with cattle husbandry) were infected with Mycobacterium tuberculosis including 6.8% were resistant to one or two anti‐TB drugs. No *Mycobacterium* spp. was found in cattle. Detection of *Mycobacterium tuberculosis* among workers in the live cattle market poses possible risk of transmission of tuberculosis to people in the live cattle market.

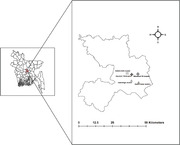

## 1 INTRODUCTION

Tuberculosis (TB) is one of the world's deadliest diseases, with an estimated 10.6 million new cases and 1.6 million deaths in 2021 (Global Tuberculosis Report, 2021). Approximately 6% of new TB cases occurred in the World Health Organization (WHO) South‐East Asia Region in 2021 (Global Tuberculosis Report, 2021). Among the six WHO regions, the South‐East Asian Region had the highest TB incidence rate (234/100,000/year) in 2021 (Global Tuberculosis Report, 2021). Likewise, TB is one of the important public health problems in Bangladesh (Zaman, 2010). According to the WHO, Bangladesh is one of 30 countries with a high TB burden worldwide, with an estimated annual incidence rate of 221/100,000/year in 2021 (Global Tuberculosis Report, 2021).

Zoonotic TB is a form of TB in people caused primarily by *Mycobacterium bovis*, which belongs to the *Mycobacterium tuberculosis* complex (MTC). Other potential MTC strains that cause zoonotic TB in Asia include *Mycobacterium orygis* identified from hospitalised TB patients in India (Duffy et al., 2020), dairy cattle, and rhesus macaque in Bangladesh (Rahim et al., 2017). *Mycobacterium bovis* causes chronic TB in cattle and other mammalian hosts (LoBue et al., 2010), affecting the production of milk and meat for consumption in these animals (Gutiérrez et al., 1995; Higino et al., 2011). Humans can become infected with *Mycobacterium bovis* through direct contact with infected animals, either by airborne transmission or by consuming infected unpasteurised milk or meat products (Grange & Yates, 1994). People in specific occupations such as farmers, veterinarians, slaughterhouse workers, and butchers have an occupational risk for zoonotic TB (Adesokan et al., 2012; Robinson et al., 1988).

Globally there were an estimated 140,000 new human TB cases and 11,400 deaths caused by *Mycobacterium bovis* in 2020 (Global Tuberculosis Report, 2020). In South‐East Asia, the estimated number of zoonotic TB cases was 43,400 in 2020 (Global Tuberculosis Report, 2020, Ramos et al., 2020). The actual burden of zoonotic TB is unknown due to the lack of surveillance data in most low‐income countries (Müller et al., 2013; Olea‐Popelka et al., 2016; Wedlock et al., 2002).

People occupationally exposed to cattle, such as livestock farmers and abattoir workers, are at a higher risk of contracting TB (Khattak et al., 2016). Evidence suggests that 2% of livestock farmers and 25% of abattoir workers were infected with TB in Pakistan (Khattak et al., 2016). Another study reported high prevalence (76%) of latent TB infection among dairy farm workers (tested by tuberculin skin test) exposed to cattle in Mexico (Torres‐Gonzalez et al., 2013). There is evidence that 10% of livestock traders were infected with TB in Nigeria (Hambolu et al., 2013). Likewise, about 2% of slaughterhouse workers were reported to be infected with TB in Iraq (Al‐Thwani & Al‐Mashhadani, 2016).

Bovine tuberculosis is an important cattle health problem in low‐and middle‐income countries. Globally, an estimated 7.4% of cattle had positive reactions to tuberculin skin tests (Grace et al., 2012). The tuberculin skin test (TST) is the primary screening test used to identify cattle infected with bovine tuberculosis. The screening test is not likely perfect, considering its sensitivity (the ability of the test correctly identify animals with the bovine tuberculosis) and specificity (the ability of the test to correctly identify animals without bovine tuberculosis) (Praud et al., 2015). The sensitivity and specificity of the TST can be influenced by a variety of factors, including the strain of the bacteria causing bovine tuberculosis, purified protein derivative products, the subjective injection, measurement ability of the test performer, the age and immune status of the animal being tested (De La Rua‐Domenech et al., 2006; Kleeberg, 1960; Snider, 1982; Schiller et al., 2010). Bovine tuberculosis surveillance is rare in in Bangladesh. However, several cross‐sectional studies reported the prevalence of bovine tuberculosis in cattle, ranging from 3% to 28% according to TST in different geographical areas in Bangladesh (Biswas et al., 2017; Islam et al., 2021; Islam et al., 2020; Islam et al., 2007; Mahmud et al., 2014; Pharo et al., 1981; Samad & Rahman, 1986).

Several risk factors make people vulnerable to zoonotic TB infections in Bangladesh. For example, 50% of the country's population is associated with some form of livestock production (Department of Livestock Services, 2014), and an extremely high density of people (1252/km^2^) (Bangladesh Population, 2016) and livestock (145 domestic ruminants/km^2^) (Ullah et al., 2015). This high animal–human density creates opportunities for close contact between TB‐infected livestock and humans during handling of animals and production of animal products, specifically milking, herding cattle and goats, slaughtering, handling skins and hides, moving cow dung, and plastering walls with dung or mud (Grace et al., 2012). However, there is no published data on the frequency of TB in humans who are occupationally exposed to cattle at the human‐animal interface in Bangladesh. We hypothesised that individuals having occupational exposure to cattle at the human‐animal interface are at high risk for contracting TB in Bangladesh.

Slaughterhouse surveillance for bovine tuberculosis in cattle is an important part of the bovine tuberculosis control program in high‐income countries (Humphrey et al., 2014; Kaneene et al., 2006; Pascual‐Linaza et al., 2017). Similarly, slaughterhouse‐based surveillance has been conducted for bovine tuberculosis in cattle in low‐ and middle‐income countries (Agbalaya et al., 2020; Carneiro et al., 2019; Demelash et al., 2009; Egbe et al., 2016; Ejeh et al., 2013; Igbokwe et al., 2001; Nalapa et al., 2017). A slaughterhouse could be an excellent setting to study the frequency and types of TB infections in cattle in Bangladesh. To our best knowledge, there is a lack of effective bovine tuberculosis surveillance utilising molecular diagnostic techniques such as polymerase chain reaction (PCR), spoligotyping, and conventional culture of *Mycobacteria bovis* in Bangladesh.

The objective of this study's was to determine the frequency of TB in individuals with occupational exposure to cattle and to detect *Mycobacterium bovis* in cattle in slaughterhouses. Furthermore, we also examined TB isolates for drug (anti‐mycobacterial drugs) susceptibility patterns. The study findings could provide a preliminary understanding of the risk of TB among individuals with occupational exposure to cattle and the occurrence of *Mycobacterium bovis* in cattle in Bangladesh.

## 2 MATERIALS AND METHODS

### 2.1 Study settings, design and period

**FIGURE 1:**
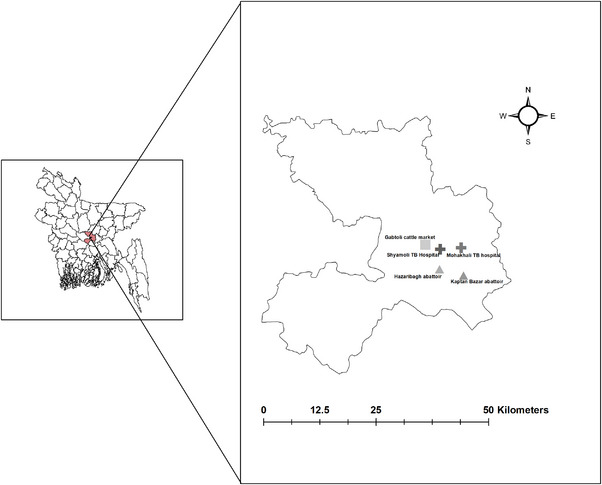
Map showing the areas where human and animal samples were collected in Bangladesh, 2014–2015.

Between August 2014 and September 2015, an observational study was conducted in two government chest disease hospitals, one cattle market, and two slaughterhouses in Dhaka (Figure 1). [Correction added on 27 June 2023, after first online publication: In the preceding sentence, the year “2014” has been added after the word “August”.] The chest disease hospitals were purposively selected where suspected TB cases were visited for TB diagnosis and treatment from neighbouring areas of Dhaka. Similarly, the cattle market was purposively selected, where cattle workers were occupationally exposed to cattle at the market during routine cattle market days. Likewise, the slaughterhouses were purposively selected where large numbers of cattle were slaughtered (on average, 40 cattle were slaughtered at the two slaughterhouses daily during the study period).

#### 2.1.1 Sampling strategy for humans

To sample suspected human TB cases, we collaborated with hospital physicians for the screening and sample collection from the suspected TB cases at the two government chest disease hospitals. We followed the guidelines of the Bangladesh National Tuberculosis Control Program (NTP) (National Tuberculosis Control Programme, 2004) for enrolling suspected TB patients. The suspected TB case was defined as a person who presents with a persistent cough for 3 weeks or more, with or without production of sputum despite the administration of a nonspecific antibiotic (National Tuberculosis Control Programme, 2004) and exposed to cattle while feeding, handling or cleaning at cattle farm in any part of their life >2 weeks before the interview. After obtaining written informed consent, a medical technologist collected three consecutive sputum specimens from each TB‐suspected participant after oral gurgling with sterile water, and specimens were kept in labelled screw cap disposable plastic bottles. Additionally, a trained medical technologist screened cattle market workers with suspected TB (persons with a persistent cough for 3 weeks or more, with or without production of sputum) following the guidelines of the Bangladesh National Tuberculosis Control Program (NTP) for suspected TB (National Tuberculosis Control Programme, 2004) and who were exposed to cattle in the past year. Cattle market workers were considered to be exposed to cattle at the market if they had contact with them during feeding, selling meat, and cleaning market places in which cattle were sold at least 2 weeks prior to the interview. After screening cattle workers with suspected TB, medical technologists collected three consecutive sputum specimens from each participant after oral gurgling with sterile water, and specimens were kept in labelled screw cap disposable plastic bottles. Sputum samples were collected in sterile containers and stored at an appropriate temperature of 2–8°C before being transported to the Mycobacteriology Laboratory at the International Centre for Diarrhoeal Disease Research, Bangladesh (icddr,b), where they were stored at −20°C until testing.

Regarding the number of people to be selected for sputum sample collection, we assumed that cattle exposed group had a 3% prevalence of tuberculosis caused by *Mycobacterium bovis* (Adesokan et al., 2012) with a 1.5% precision, and 95% confidence level, a sample size of 497 was calculated.

#### 2.1.2 Sampling strategy for animals

We collaborated with Dhaka City Corporation South (under the Ministries of Local Government and Rural Development) to conduct the study. The field team, comprising trained veterinarians, field research assistants, visited each slaughterhouse twice a week in the early morning. A trained veterinarian assessed the overall physical status of animals using a five‐point body condition score (BCS) scale, where animals were classified as emaciated (BCS 1), thin (BCS 2), normal (BCS 3), muscular (BCS 4), and fat (BCS 5) (Katale et al., 2013; Msangi et al., 1999). The BCS score of 2 was used to screen animals for sample collection. The animals had a BCS score of 2, indicating emaciation to thinness, and they had a sunken appearance with visible ribs, hips and backbone (Edmonson et al., 1989). Veterinarians collected tissue samples that revealed visible granulomatous lesions and/or caseous masses from the lungs, liver, intestines, and lymph nodes of slaughtered cattle that had emaciated to thin body condition. The tissue samples were collected in sterile containers and stored at an appropriate temperature of 2–8°C before being transported to the Mycobacteriology Laboratory at the icddr,b where they were stored at −20°C until testing.

#### 2.1.3 Data collection

Field research assistants collected demographic information (age, sex) from human participants using a separate, structured questionnaire. Veterinarians collected demographic information (age, sex, type of breed) of sampled cattle from animal owners/abattoir worker using a structured questionnaire.

### 2.2 Laboratory testing

#### 2.2.1 Human sputum and cattle tissue processing, staining and culture

**FIGURE 2:**
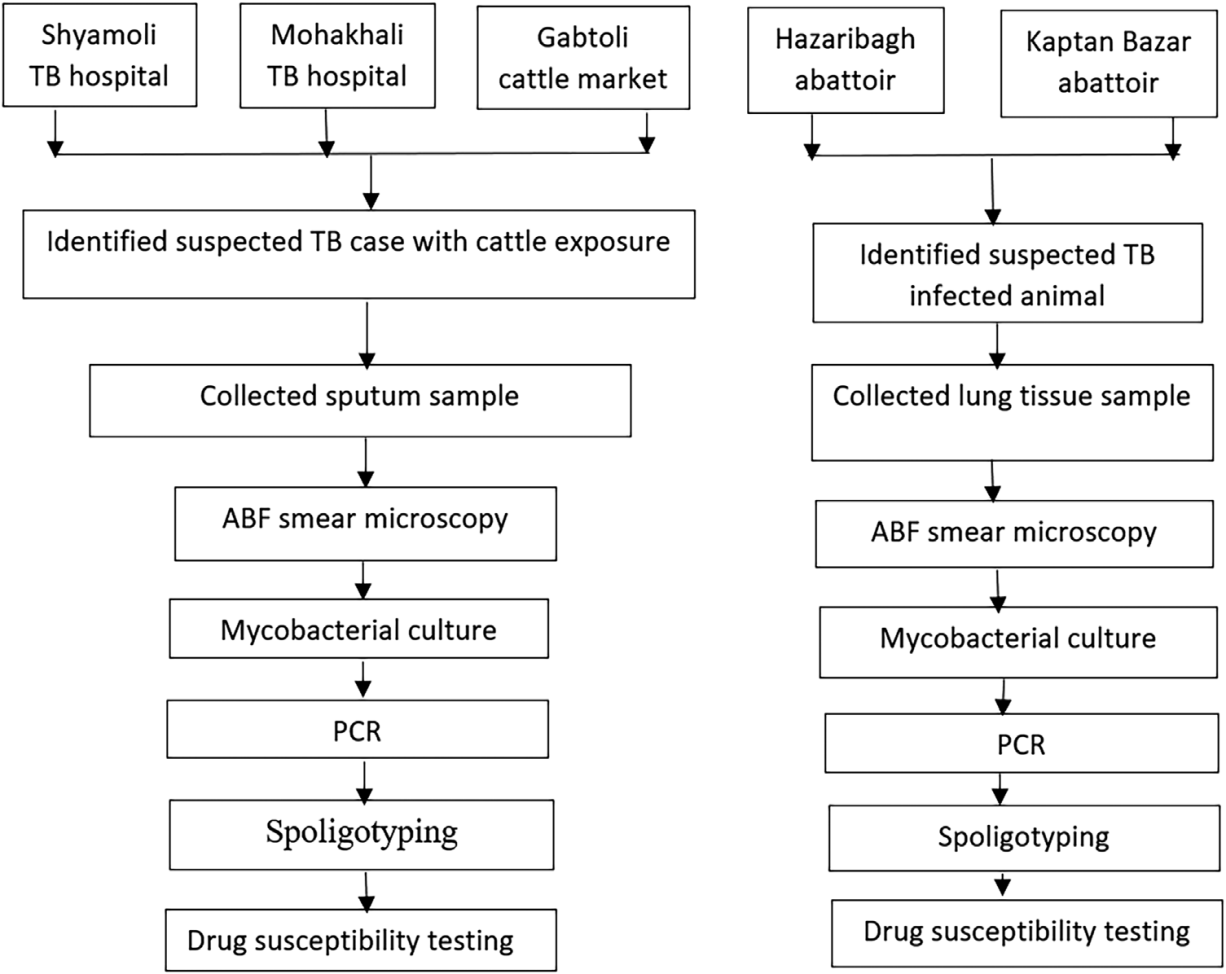
Study flowchart.

Human sputum and cattle tissue samples were obtained according to the standard operating procedures and guidelines (Petroff, 1915; Zaman et al., 2006). Human sputum specimens were digested and decontaminated (Petroff, 1915) and then inoculated on Lowenstein–Jensen (L–J) slant cultures. The L–J slants were incubated at 37°C for up to 8 weeks and visibly examined once per week for contamination and growth of *Mycobacterium spp* colonies. The cattle tissue samples were processed following the technique described earlier (Rahim et al., 2007). In brief, small pieces of tissue were sliced by a sterile surgical blade and homogenised in sterile 5 mL phosphate‐buffered saline (PBS) using a tissue homogeniser in a bio‐safety cabinet. The homogenate was mixed with an additional 10 mL PBS and allowed to settle for 15 min at room temperature. Approximately 5 mL of supernatant was collected and decontaminated following Petroff's methods (Petroff, 1915). Finally, the pellet was re‐suspended in 1 mL PBS and used for culture on two Lowenstein–Jensen (L–J) slants with and without sodium pyruvate. The processed samples were inoculated simultaneously in two L–J slants; one with glycerol but no sodium pyruvate for the growth of *M. tuberculosis* and another L–J contained sodium pyruvate but not glycerol for the growth of *Mycobacterium bovis* (Corner & Nicolacopoulos, 1988). Human sputum and cattle tissue samples were considered culture negative if no visible mycobacterium colonies were seen on L–J slants following 8 weeks of incubation at 37°C (Banu et al., 2012). Human sputum and cattle tissue specimens were also examined for acid‐fast bacilli (AFB) using Ziehl–Neelsen (Z–N) staining and light microscopy (Figure 2) following methods described earlier (Organization, 1998).

### 2.3 Anti‐mycobacterial drug susceptibility test

Drug susceptibility testing (DST) was done on culture‐positive isolates following conventional proportion susceptibility methods (Canetti et al., 1969). MTC isolates were tested for susceptibility to isoniazid, rifampicin, ethambutol, and streptomycin according to the method described by Canetti et al. (1969). An isolate was considered to be resistant to a given drug when any growth of 1% or more above the control was observed in a quadrant plate containing the drug (Banu et al., 2012). Pyrazinamide (PZA) susceptibility testing was also done on all MTC isolates using the methods described by Rahman et al. (2017).

#### 2.3.1 *Mycobacterium tuberculosis*‐specific region of difference 9 (RD9) analysis

Polymerase chain reaction (PCR)‐based on RD9 deletion analysis was performed using forward: 5´ CGATGGTCAACACCACTACG‐3´ and reverse: 5´‐CTGGACCTCGATGACCACTC‐3´ primer sets, according to previously described methods (Brosch et al., 2002) to detect *Mycobacterium tuberculosis*.

#### 2.3.2 Spoligotyping

Spoligotyping was performed on MTC‐positive isolates according to the standard protocol described by Kamerbeek et al. (1997). The presence or absence of 43 variable spacers in the direct repeat region of MTB was determined using DRa (5′‐GGTTTTGGGTCTGACGAC‐3′, 5′‐biotinylated) and DRb (5′‐CCGAGAGGGGACGGAAAC‐3′) primers. Each reaction mixture was prepared in a total volume of 20.0 μL, containing 2.0 μL of 10× Super Tth buffer, 0.2 mM of dNTP mix, 1.6 μM of each forward and reverse primers, 0.2 unit of Taq polymerase and 2.0 μL of template DNA PCR amplification in a Veriti 96 well Thermal Cycler (Applied Biosystems) with initial denaturation at 96°C for 3 min followed by 35 cycles of denaturation at 96°C for 1 min, annealing at 55°C for 1 min, and extension at 72°C for 1 min; this was followed by a final extension at 72°C for 5 min.

The resulting amplicons were hybridised with a commercially available membrane (Isogen Bioscience BV, Bilthoven, the Netherlands). The membrane contains 43 covalently linked synthetic oligonucleotides corresponding to 43 spacers arranged in parallel rows. Hybridised patterns were detected by Enhanced Chemiluminescence (ECL) (Amersham, UK). Detected bands were converted to 43 binary codes, and spoligotype data were analysed using SITVITWEB, an online tool of the Institute Pasteur de Guadeloupe (http://www.pasteur‐guadeloupe.fr:8081/SITVIT_ONLINE/).

#### 2.3.3 Quality assurance of the testing

All the procedures, including sample preparation, decontamination, and culture, were done in the Mycobacteriology Laboratory of icddr,b (https://www.icddrb.org/). All the procedures were done in aseptic conditions using a class II A2 Biosafety cabinet and air‐locked centrifuge to avoid any cross‐contamination or aerosol generation. Also, laboratory personnel wore appropriate personal protective equipment while processing the specimens. Moreover, we used appropriate controls included in the L–J slant culture method and the Z–N staining. During inoculation into L–J slant culture, we always used PBS as a negative control and H37Rv as a positive control. For Z–N staining, we referenced known graded positive and negative slides.

#### 2.3.4 Data analysis

We summarised the categorical variables using frequencies and percentages. We reported the means with standard deviations of continuous variables for symmetric distributions and medians with interquartile ranges (IQRs) for asymmetric distributions. We calculated the overall proportion of TB (based on positive AFB staining and/or culture results) in humans and cattle with 95% confidence intervals (CI). Statistical analyses were performed using Stata version 13 (Stata Corporation, College Station, TX, USA).

## 3 RESULTS

A total of 412 humans sputum samples were analysed in this study (Table 1). Of the 412, 382 and 30 samples were collected from the hospitals and cattle market, respectively. The median age of human participants was 35 (IQR: 25–50) years, 75% were male (Table 1), and the median body mass index (BMI) was 17.9 (IQR: 16.7–19.6). Approximately two‐thirds of the participants were aged 16–45 years (Table 1). Most participants (85%) reported receiving the Bacillus Calmette‐Guerin (BCG) vaccine in the past. All human participants reported that they had been exposed to cattle the previous year.

**TABLE 1:** Frequency of Mycobacterium tuberculosis complex (MTC) among human participants by age, sex and location, Bangladesh, 2014–2015 (*N* = 412).

Characteristics	No. of participants examined (%)	AFB‐positive frequency (%)	MTC culture‐positive frequency (%)	Both AFB, MTC culture and PCR positive frequency (%)
*Study site*				
Shaymoli^a^	285 (69)	19 (6.6)	31 (10.8)	18 (78.3)
NIDCH	97 (24)	3 (3.0)	10 (10.3)	3 (13)
Cattle market	30 (7)	3 (10)	3 (10)	2 (8.7)
*Sex*				
Male	309 (75)	18 (5.8)	31 (10)	16 (70)
Female	103 (25)	7 (6.8)	13 (12.6)	7 (30)
*Age group (year)*				
<15	41(10)	0 (0)	2 (4.8)	0 (0)
16–45	279 (68)	20 (7.1)	33 (11.4)	18 (78)
>45	92 (22)	5 (5.4)	9 (9.7)	5 (22)

Abbreviations: NIDCH, National Institute of Chest Disease and Hospital; AFB, acid‐fast bacilli (AFB) smear‐positive.

^a^ Shamoly Chest Disease Hospital.

Twenty‐five (6.1%, 95% CI: 4–9%) of the 412 humans had AFB smear‐positive results (Table 1). Of the 412 humans, 44 were mycobacterial culture positive for MTC (11%, 95% CI: 8–14%) (Table 2). Twenty‐three of the 412 people tested positive for both AFB, mycobacterial culture and RD9 PCR positive for MTC (5.6%, 95% CI: 3.6–8.2%) (Table 1).

**TABLE 2:** Spoligotyping patterns of *Mycobacterium tuberculosis* in human isolates in Bangladesh (*N* = 44).

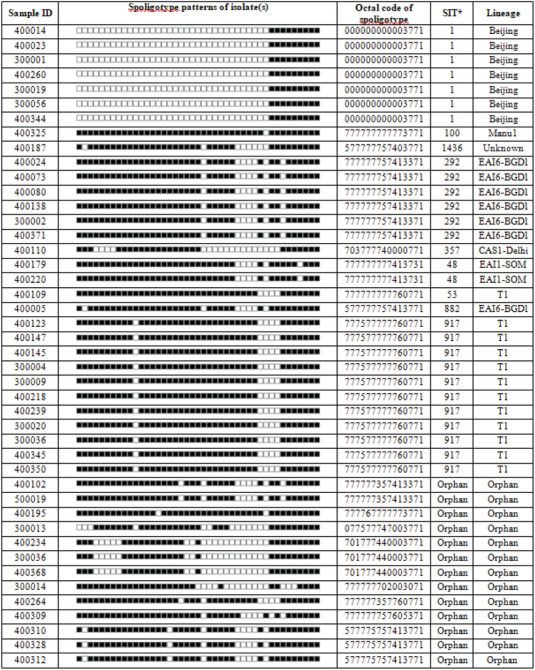

^a^ SIT, shared‐type‐number of the SpolDB4/SITVIT database.

Based on RD9 PCR, all 44 MTC isolates were identified as *Mycobacterium tuberculosis*. Among the 44 MTC culture positive, the most common circulating spoligotypes were T1 (12; 27%), followed by Beijing (7; 16%) and East African Indian EAI 6‐BGD1 (7; 16%) strains (Table 2). We also found some less frequent spoligotypes, such as Central Asian Strain 1‐Delhi (CAS 1‐Delhi) (1; 2%), Manu 1 (1; 2%), and EAI1‐SOM (1; 2%). The specific spoligotype pattern could not be determined for the remaining 15 (34%) culture‐positive cases designated as an Orphan or unknown lineage (Table 2). We also detected three (10%; 3/30) cattle market workers were infected with *Mycobacterium tuberculosis* (Table 1). Drug susceptibility testing showed that 41 (93%) of the 44 MTC isolates were susceptible to all anti‐TB drugs (isoniazid, rifampicin, ethambutol, streptomycin and pyrazinamide) tested. However, 6.8% (3/44) *Mycobacterium tuberculosis* cases were resistant to one or two anti‐TB drugs. Among three anti‐TB drugs resistant cases, one case was resistant to streptomycin and pyrazinamide (52 years, male), a second case was resistant to streptomycin alone (55 years, male), and another case was resistant to pyrazinamide alone (30 years, male). All three drug‐resistant cases were derived from one of the chest disease hospital sites.

A total of 169 cattle tissue samples were obtained for analysis (Table 3). The median age of the cattle was 36 months (IQR: 25–48 months) (Table 3). The median BCS of cattle was 2 (IQR: 2–2.5) (Table 3). Most of the cattle (76%) were female (Table 3), and the majority of the cattle (67%) included in the study were pure indigenous breeds. During this study, no cattle tissue samples had granulomas lesions. All the cattle tissue samples were neither AFB nor culture positive for MTC.

**TABLE 3:** Demographic characteristics of sampled cattle in Bangladesh, 2014–2015 (*N* = 169).

Variable	Cattle (*N* = 169)
Age in month, median (IQR)	36 (25–48)
Sex, frequency (%)	
Female	129 (76)
Breed frequency (%)	
Indigenous	116 (67)
Body condition score, median, IQR	2 (2–2.5)

## 4 DISCUSSION

Our findings provided a preliminary insight about TB among people exposed to cattle and those working in slaughterhouses in Dhaka city. The study detected pulmonary *Mycobacterium tuberculosis* among 11% of humans who were presented to the chest disease hospitals with symptoms of TB, as well as 10% of workers in the cattle market. The proportion of *Mycobacterium tuberculosis* infection among cattle workers was higher (10%) in this study compared to another study (6.67%) conducted in Bangladesh (Rahman et al., 2015). These findings have public health importance. For example, if infected cattle market workers remain undetected and untreated, this may pose a risk of spreading of *Mycobacterium tuberculosis* via droplets to buyers and fellow workers in the cattle market, where crowded and close contact is common. We would recommend a periodic TB screening program for cattle market workers and conducting a health education campaign about transmission, diagnosis, treatment, and prevention of TB.

Our study found that two‐thirds of *Mycobacterium tuberculosis* cases occurred among the 16–45 years age group in humans, which is a higher proportion compared to the report of the National Tuberculosis Control Program 2017 (63% among 15–54 years age group), but it was not statistically significant. This study also provided information on the spoligotype and drug susceptibility pattern of *Mycobacterium tuberculosis* isolates among humans.

Our study did not detect TB cases caused by *Mycobacterium bovis* among humans. It was possible that they had never been exposed to *Mycobacterium bovis‐*infected animals or contaminated, unpasteurised animal products in the year prior to sample collection. It could also explain that all *Mycobacterium tuberculosis* isolates were identified from the sputum samples of suspected pulmonary TB (PTB) cases, which could have decreased the likelihood of detection of *Mycobacterium bovis* in this study. Because *Mycobacterium bovis* is associated with extrapulmonary TB, which limit the ability to detect *Mycobacterium bovis* in sputum sample. Studies suggest that *Mycobacterium bovis* infections cause a higher proportion of extra pulmonary TB (EPTB) compared to PTB cases in humans (9.4% vs. 2%) (Cosivi, 1998; Prasad et al., 2005; Rasolofo‐Razanamparany et al., 1999). Also, other potential mycobacterium species, such as *Mycobacterium orygis*, cause zoonotic TB infections in humans in India (Duffy et al., 2020) and dairy cattle, and rhesus macaque in Bangladesh (Rahim et al., 2017) were not tested in our study samples. A future study could target EPTB cases to detect *Mycobacterium bovis* infection in individuals using whole genome sequencing (Duffy et al., 2020) or more comprehensive PCR (Duffy et al., 2020) in Bangladesh.

The study was not able to detect *Mycobacterium bovis* in cattle in the slaughterhouses investigated, given negative bacteriological and molecular assay test results. The absence of *Mycobacterium bovis* in cattle might be explained by the fact that 67% of sampled cattle were an indigenous breed that is naturally less susceptible to *Mycobacterium bovis* infection than nonindigenous breeds of cattle (Liston & Soparkar, 1917; Soparkar, 1926; Vordermeier et al., 2012). In fact, Bangladeshi indigenous cattle are still more prevalent (85%) than the cross‐bred cattle (15%) (Hamid et al., 2017). Previous studies have indicated that *Mycobacterium bovis* infection is more common among nonindigenous than indigenous cattle breeds in Bangladesh (Islam et al., 2007; Hossain et al., 2012; Rahim et al., 2007; Samad & Rahman, 1986). Prior studies using a tuberculin skin test also reported that the prevalence of *Mycobacterium bovis* among cattle in Bangladesh ranges from 2.1% to 34% (Islam et al., 2010; Samad & Rahman, 1986; Uddin et al., 2014). Moreover, the study with fewer cattle sampled (*n* = 169) could have limited our ability to detect the *Mycobacterium bovis* in the slaughterhouse settings in Dhaka city. The study also observed that most of the cattle had good BCS >2 when brought into the slaughterhouse for slaughtering in Dhaka City. Thus, the slaughterhouse may not be an ideal setting to investigate tuberculosis caused by *Mycobacterium bovis* in the context of Bangladesh. Because the study found that most of the cattle belonged to indigenous breed, which is less susceptible to *Mycobacterium bovis* as referenced earlier.

This study had a number of limitations. First, we collected samples from humans presenting for diagnosis and TB care in the two main chest disease hospitals and a single cattle market, all located in Dhaka. All human patients had symptoms of PTB and collected sputum samples, which may not be a true representation of the human population exposed to cattle infected with *Mycobacterium bovis* in Bangladesh. So, future study should target extrapulmonary TB cases to investigate the prevalence of *Mycobacterium bovis* in humans in Bangladesh. Second, we were unable to collect optimum samples from nonindigenous breeds (cross or exotic breed) of cattle from the slaughterhouses, which would have severely limited our ability to examine differences in the occurrence of *Mycobacterium bovis* between indigenous and nonindigenous breeds of cattle if any infections with *Mycobacterium bovis* had been identified. During the study inception, we assumed that slaughterhouses would have been ideal study settings to examine the occurrence of *Mycobacterium bovis* in indigenous and nonindigenous breeds of cattle. However, the study found that 67% of the cattle were indigenous breeds in slaughterhouses. Future epidemiologic studies should emphasise in geographic areas/settings where higher proportions of nonindigenous breeds of cattle farms are present to assess the risk of *Mycobacterium bovis* infections. Fourth, we selected cattle for sampling based solely on low body condition score (emaciated to thin), which could be a less sensitive marker, thus, limiting our detection of *Mycobacterium bovis* among studied cattle*. Mycobacterium bovis* infection in cattle is a chronic condition with long‐term progression. Future studies should be considered to include specific clinical criteria (such as a chronic, moist cough) along with older cattle (≥4 years) as inclusion criteria for routine cattle sampling for bovine tuberculosis. Besides, interpret the absence of *Mycobacterium bovis* (negative findings) in cattle with caution, and consider the number of cattle that were not large enough. In addition, most of the sampled cattle were indigenous breeds and the median age of cattle was three years during the study.

In conclusion, we did not detect any TB case caused by *Mycobacterium bovis* in humans during the study. However, we detected TB cases caused by *Mycobacterium tuberculosis* in all human participants, including cattle market workers. Detection of *Mycobacterium tuberculosis* among cattle market workers pose a potential public health risk of TB spreading via droplet. Public health measures, including routine TB screening and awareness programs in people occupationally exposed to cattle, could help to control TB in humans in Bangladesh. Moreover, these findings could persuade the relevant stakeholders to design systemic targeted surveillance or epidemiological research that, for example, quantify and observes the level of cattle exposure among high‐risk individuals (workers in cattle market, slaughterhouse workers, veterinarians) and those that have occupational exposure to cattle for aiding a TB prevention and control in high‐risk individuals at high‐risk settings. Also, this study could persuade the relevant stakeholders to develop preventive health education messages to decrease the risk of TB, such as wearing a face mask (Philip et al., 2002) while visiting and working in a live cattle market in Bangladesh. Finally, diagnosing zoonotic TB is difficult because of the variation in media and the nature of the organisms (Kock et al., 2021). New and easy‐to‐use diagnostic tests, such as interferon‐γ assays are needed to be developed and validated to detect zoonotic TB in animals and humans, and thus contribute to the eradication of TB (Kock et al., 2021).

## AUTHOR CONTRIBUTIONS

Shamim Sarkar: Conceptualisation, data curation, formal analysis, investigation, methodology, project administration, writing – original draft. Najmul Haider: conceptualisation, investigation, methodology, writing – review & editing. Md Ariful Islam: conceptualisation, investigation, methodology, writing – review & editing. Muhammad Belal Hossain: investigation, writing – review & editing. Kamal Hossain: methodology, writing – review & editing. Mohammad Khaja Mafij Uddin: investigation, writing – review & editing. Arafatur Rahman: investigation, writing – review & editing. Syed Sayeem Uddin Ahmed: writing – review & editing. Sayera Banu: writing – review & editing. Zeaur Rahim: investigation, writing – review & editing. James D Heffelfinger: methodology, writing – review & editing. Nord Zeidner: methodology, supervision, writing – review & editing.

## CONFLICT OF INTEREST STATEMENT

The authors declare no conflict of interest in doing these studies.

### DISCLAIMERS

The findings and conclusions in this report are those of the authors and do not necessarily represent the views of the Centers for Disease Control and Prevention or the Agency for Toxic Substances and Disease Registry.

### ETHICS STATEMENT

The study protocol was reviewed and approved by the Ethical Review Committee and Animal Experimentation Ethical Committee of the International Centre for Diarrhoeal Disease Research, Bangladesh(icddr,b) (Protocol number: PR‐14007). All human participants provided written informed consent before collecting their sputum and data.

### PEER REVIEW

The peer review history for this article is available at https://publons.com/publon/10.1002/vms3.1178.

## Data Availability

The data that support the findings of this study are available from the corresponding author upon reasonable request.
